# Sheep and goat pathogen database: a pathogen data integration and analysis database of sheep and goat infectious diseases

**DOI:** 10.3389/fmicb.2023.1299303

**Published:** 2024-01-12

**Authors:** Haoju Pan, Zizhuo Jiao, Hong Li, Suya Li, Le Xu, Shiyuan Li, Yong Meng, Yujing Fu, Taoyu Chen, Qiaoling Chen, Si Chen, Li Du, Churiga Man, Fengyang Wang, Hongyan Gao

**Affiliations:** Hainan Key Laboratory of Tropical Animal Reproduction and Breeding and Epidemic Disease Research, Animal Genetic Engineering Key Laboratory of Haikou, School of Tropical Agriculture and Forestry, Hainan University, Haikou, China

**Keywords:** sheep and goat infectious disease, pathogen, database, resource integration, public health

## Abstract

The prevalence of infectious diseases in sheep and goats has a significant impact on the development of the sheep and goat industry and public health security. The identification and analysis of pathogens are crucial for infectious disease research; however, existing databases pay little attention to sheep and goat diseases, and pathogen data are relatively scattered. Therefore, the effective integration, analysis and visualization of these data will help us conduct in-depth research on sheep and goat infectious diseases and promote the formulation of disease prevention and control strategies. This article considered the pathogens of 44 infectious diseases in sheep and goats as the main research objects and collected and downloaded relevant scientific literature, pathogen genomes, pathogen transcriptomes, pathogen occurrence records, and other data. The C# programming language and an SQL Server database were used to construct and realize the functions of the Sheep and Goat Pathogen Database (SGPD) within a B/S architecture based on the ASP.NET platform. The SGPD mainly provides an integrated platform for sheep and goat pathogen data retrieval, auxiliary analysis, and user upload, including several functionalities: (1) a Disease Introduction module that queries basic information regarding the 44 recorded sheep and goat infectious diseases, such as epidemiology, clinical characteristics, diagnostic criteria, and prevention and control measures; (2) an Omics Information module that allows users to query and download the genome and transcriptome data related to the pathogens of sheep and goat infectious disease, and provide sequence alignment functionality; (3) a Pathogen Structure module that enables users to view electron micrographs of pathogen structure and tissue sections related to sheep and goat disease from publicly published research; (4) a Literature Search module based on the “Pathogen Dictionary” search strategy that facilitates searches for published research related to pathogens of infectious disease; (5) a Science Popularization module that allows users to view popular science materials related to sheep and goat infectious diseases; and (6) a Public Health module that allows users to query the risk factors of zoonotic disease transmission and the corresponding related literature, and realize the visualization of pathogen distribution. The SGPD is a specialized sheep and goat pathogen information database that provides comprehensive resources and technical support for sheep and goat infectious disease research, prevention, and control.

## 1 Introduction

The diversity of pathogens transmitted by sheep and goats is continuously increasing (Galante et al., 2019). Severe infectious diseases, persistent morbidity and mortality, and jeopardization caused by multiple pathogens are detrimental to sheep and goat health. The increased costs associated with vaccination and disease treatment can also affect sheep and goat breeding (Simões et al., 2021). The direct economic losses in provinces involving sheep and goats infected with *Brucella* can reach up to 1.8–2.04 billion RMB according to a survey conducted by the China Animal Health and Epidemiology Center on the main sheep-producing areas in China (Gao et al., 2022). In addition, zoonoses such as anthrax, foot-and-mouth disease, and toxoplasmosis pose serious threats to animal husbandry and public health security (Cao X. et al., 2018; Stelzer et al., 2019; Mugezi et al., 2020; Stella et al., 2020; Zhang et al., 2022; Omodo et al., 2023). Therefore, there is an urgent need to prevent and control infectious diseases in sheep and goats. An in-depth study of the epidemic pathogen provides an effective breakthrough for rapid and accurate diagnosis and treatment to successfully curb the outbreak of sheep and goat epidemics. For example, Ouchene et al. (2023) investigated the seropositivity rate and pathogenic risk factors for *Toxoplasma gondii* infection in Algerian sheep and identified the risk factors for pathogen transmission to provide an effective basis for the rational prevention of toxoplasmosis in sheep. Hawwas et al. (2022) collected fecal samples from sheep, goats, and humans in the Suez Canal area and combined the results with the prevalence and bacterial serotypes to show that sick sheep and goats are an important source of highly virulent *Salmonella* infection in humans. In addition, the development and evolution of sequencing tools have provided technical support for the in-depth study of pathogens and discovery of new strains or virus subtypes (Lange et al., 2023). Therefore, the effective integration and utilization of a large amount of pathogen data are crucial to establish new intervention strategies for sheep and goat infectious diseases.

Current pathogen data and related analysis results are widely distributed in various types of data resources. The most commonly used sequence data management systems for sheep and goat pathogens include the National Center for Biotechnology Information (NCBI), DNA Data Bank of Japan (DDBJ), and European Molecular Biology Laboratory (EMBL) databases. There are also pathogen sequence databases for specialized species, such as a web-based database platform for swine pathogens (Wang et al., 2020). Platforms that can carry information about infectious diseases and pathogens were widely used in life sciences. The database of zoonotic and vector-borne viruses (ZOVER) built by Zhou et al. (2022) collected bat and rodent-related virus information and provided online visualization tools for comparative analysis. The microbial pathogen diagnostic methods database (MicrobPad MD) developed by Han et al. (2013) provides comprehensive information on pathogen-related molecular diagnostic techniques, targets, primers/probes, and detection procedures. Leandro et al. (2022) developed an early warning system for arbovirus transmission that can predict the epidemic situation of dengue outbreaks with high precision. Open information and resource sharing are trending in the era of big data. The integration of different types of pathogen data is important to enhance data reusability and accessibility. However, previous studies have not developed a specialized database of sheep and goat pathogen information.

This study designed and developed a pathogen information database of sheep and goat infectious diseases (Sheep and Goat Pathogen Database, SGPD) by collecting and sorting data from relevant scientific databases, literature, and monographs. This study aimed to establish a “one-click query” information retrieval and analysis platform to achieve effective sharing and reuse of data. It is expected to provide fast and accurate pathogen information retrieval tools for veterinarians and researchers and delivers a theoretical foundation and technical support for in-depth research on sheep and goat infectious diseases. This study will provide a reference for the development of pathogen databases, and the effective integration and analysis of pathogen data will have important practical significance.

## 2 Materials and methods

### 2.1 Data collection and processing

We first reviewed and collected data from online sources containing infectious disease and pathogen data, and identified specific databases (including NCBI, DDBJ, and EMBL) as candidate sources. We then conducted interviews with academics working on sheep and goat pathogens and identified some high-level findings, such as the purposes using the data, the types of data that should be contained in the database, and how users can obtain pathogen-related information. These insights were used to generate a user-centric dataset of sheep and goat pathogens.

Currently, there is information on 44 pathogens in the SGPD database (Supplementary Table 1), and the stored data types and sources are as follows: (1) literature information: sheep and goat infectious disease-related literature was downloaded from search engines such as PubMed, Web of Science, and China National Knowledge Infrastructure. Then, basic fundamental infectious disease information was obtained (including introduction, epidemiology, clinical characteristics, diagnostic points, control measures, and so on), relevant omics data, and pathogenic structure from a large amount of literature; (2) genome and transcriptome data: the reference genome and transcriptome data of the pathogens corresponding to sheep and goat infectious diseases was obtained from biological databases such as NCBI, DDBJ, EMBL; (3) infectious disease outbreak data was derived from published literature and official websites of the Office International Des Epizooties (OIE), the Food and Agriculture Organization of the United Nations, and so on; and (4) multimedia files: contain videos and pictures related to sheep and goat infectious diseases, original popular science animations from our laboratory team, and links to related scientific research websites.

### 2.2 Development of web applications

A major challenge for scientific researchers is integrating data from disparate sources into a unified dataset. Our survey included several major data types in the database: pathogens, pathogen genomes, host transcriptomes during infection, disease outbreaks, and related literature. We focused on detailed UI elements and paid more attention to page layouts, information layouts, and interactions with different pathogen data to integrate the data obtained from various sources during the development of the SGPB database.

**FIGURE 1:**
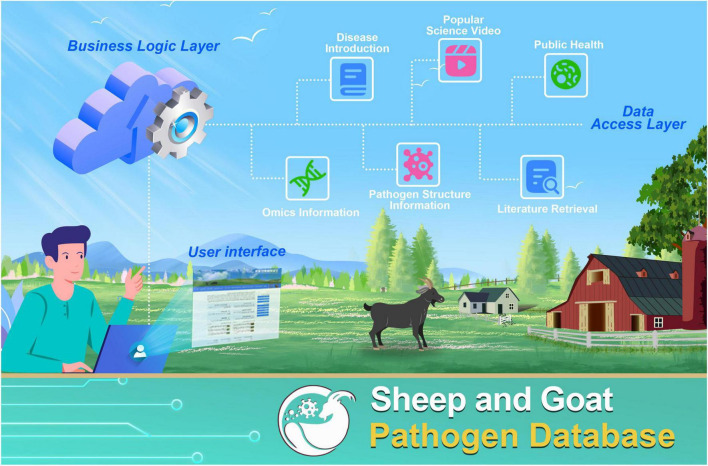
Schematic diagram of the Sheep and Goat Pathogen Database.

The SGPD database was designed and implemented based on the Asp.NET platform. All data were organized in a publicly available SQL Server database, and the frontend was a web interface based on HTML, CSS, JavaScript, and C# programming languages. The SGPD web server consists of a three-tiered architecture model: a user interface, processing logic tier, and database tier to realize data interaction between the browser and server. The main framework, interaction, and functional modules of the SGPD are shown in Figure 1.

## 3 Results

**FIGURE 2:**
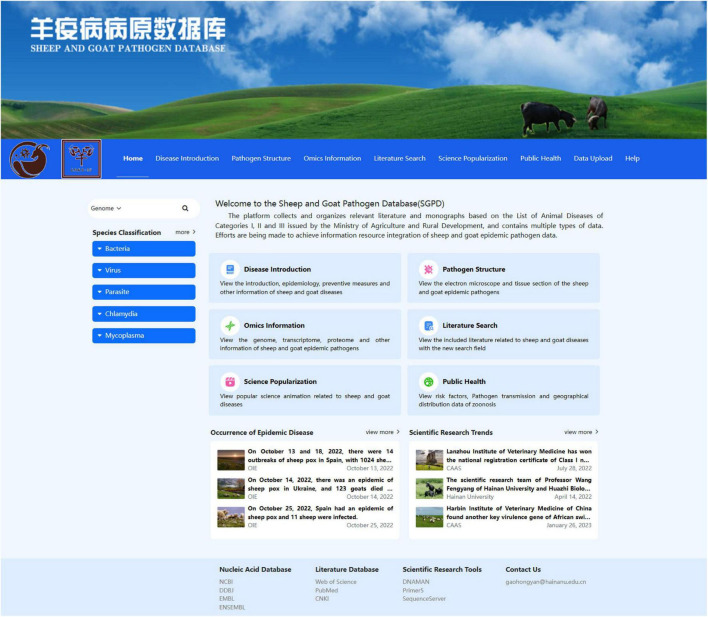
Home page of the Sheep and Goat pathogen database.

The SGPD database mainly provides a platform for sheep and goat pathogen data retrieval and auxiliary analysis and is divided into several components, including the Disease Introduction module, Pathogen Structure module, Omics Information module, Literature Search module, Science Popularization module, Public Health module, and Data Upload. The homepage of the SGPD (Figure 2) also shows a fixed navigation bar, specification classification of pathogens, six main functional modules, and other scientific information websites.

### 3.1 Data search platform for pathogen

**FIGURE 3:**
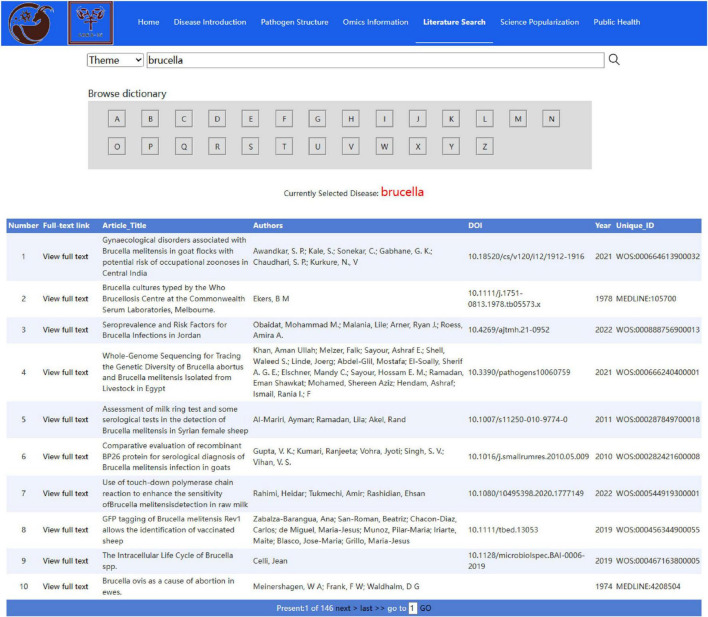
The literature search module interface.

The SGPD uses published academic papers to provide a specialized literature retrieval module related to sheep and goat pathogens. Using (pathogen name) AND ((sheep) OR (goat)) as the search field, we retrieved 55,920 articles from the Web of Science, PubMed, and other literature retrieval databases. Then, we extract and integrate effective information in the literature and works and provide users with detailed metadata (including title, author, abstract, keywords, and other information) to better describe and classify research results. The SGPD provides users with two options to retrieve objects: (1) based on the “Pathogen Dictionary” search strategy: the initial of the pathogen names are sorted according to the 26 English letters “A-Z” and the relevant literature of the target pathogen is searched by selecting the letters; (2) the subject search may include pathogen species, title, author, year and other conditions for retrieval (Figure 3). The search results provide the main information on the literature and a link to the original article, which makes it convenient for users to trace the source of the literature.

**FIGURE 4:**
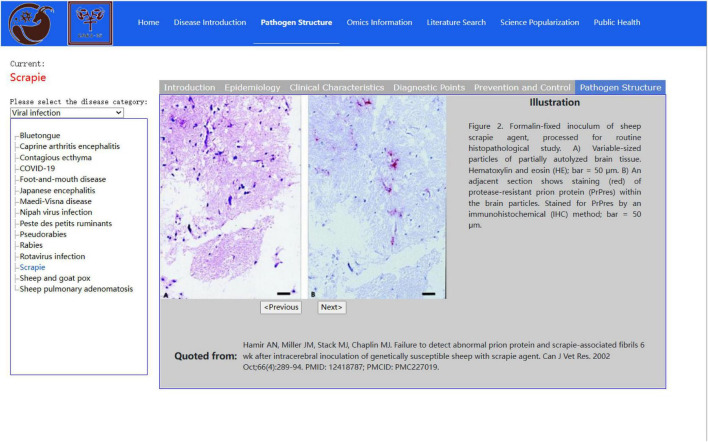
Pathogen structure interface in the disease introduction module.

The SGPD further conducts metadata management with the help of the Disease Introduction and Pathogen Structure modules, and the pathogen information is displayed to the users by categories. The Disease Introduction module provides detailed information on sheep and goat pathogens by switching between different tabs (including epidemiology, clinical characteristics, key points of diagnosis, prevention and control measures, and pathogen structure diagrams) (Figure 4). Of these, the pathogen structure diagram includes electron micrographs of pathogen structure and tissue sections, and this part of the information can also be queried from the pathogen structure module.

**FIGURE 5:**
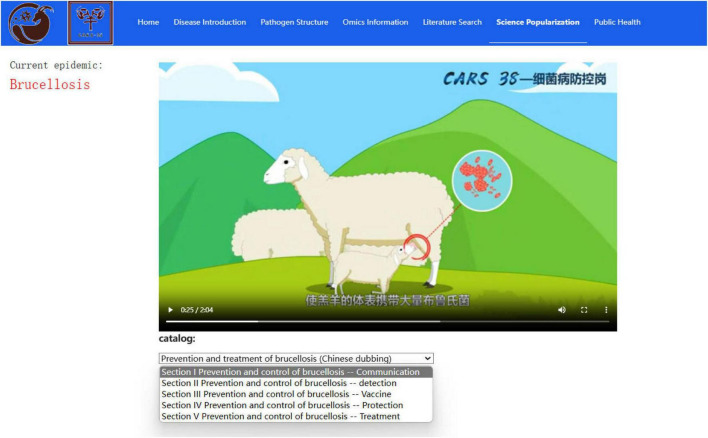
Science popularization module interface.

In addition, the Science Popularization module adopts multimedia forms to display sheep and goat infectious disease-related knowledge. This makes it easier to understand the specific content to allow users to accept natural scientific knowledge in an easy-to-understand manner. Currently, the video content includes *Brucella* transmission, detection, vaccine immunization, personnel prevention and control, and treatment (Figure 5). This laboratory is creating popular science videos of pathogens in multiple languages to expand the audience.

### 3.2 Data analysis platform for pathogens

The development of the SGPD supports pathogen data retrieval and scientific research. Furthermore, it is important to combine early warning of disease and bioinformatics with infectious disease and omics data. Therefore, SGPD provides an auxiliary analysis and visualization platform centered on pathogen data in addition to general pathogen information queries.

#### 3.2.1 Omics information module

**FIGURE 6:**
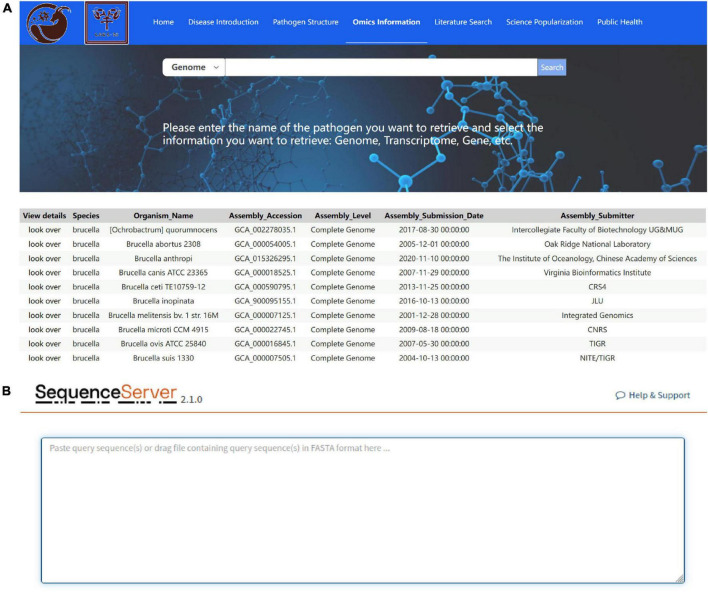
Omics Information module interface. **(A)** Retrieval results of pathogenic genome and transcriptome data; **(B)** sequence alignment performed using the SequenceServer Program to determine function.

The omics information module provides general information on the sheep and goat pathogen genome and transcriptome data. Genome data includes the species, organism name, assembly accession, assembly level, assembly submission date, and assembly submitter information of the pathogens (Figure 6A). Transcriptome data includes the article source, BioProject number, sample information, and sequencing information. Each genome and transcriptome record contains a link to the original GenBank record (if available), and the information parsed from this record can be annotated into FASTA definition lines or the metadata can be downloaded for additional analysis. In addition, the omics information module uses the SequenceServer program to perform sequence alignment of the indicated nucleic acid sequences with the sheep and goat pathogenic genome, CDS, cDNA, and ncRNA (Figure 6B).

The omics information module has the following characteristics: (1) it is a specific ovine pathogen omics retrieval tool; (2) it can analyze multiple data types, including reads, contigs, scaffolds, genes, and 16SrRNA; (3) it can save computational resources and reduce computing time; and (4) it provides comprehensive visualization results.

#### 3.2.2 Public health module

Reducing the impact of pathogens in sheep and goat requires a fundamental understanding of their transmission characteristics in the flock. The public health module aims to visualize infectious disease data and reduce the burden of infectious diseases through targeted surveillance. The public health module can efficiently apply big data technology to query the risk factors for zoonosis, transmission routes, and distribution characteristics of pathogens in time, region, and flock through the collection of a large amount of data to analyze the occurrence and development of important infectious diseases in sheep and goats.

**FIGURE 7:**
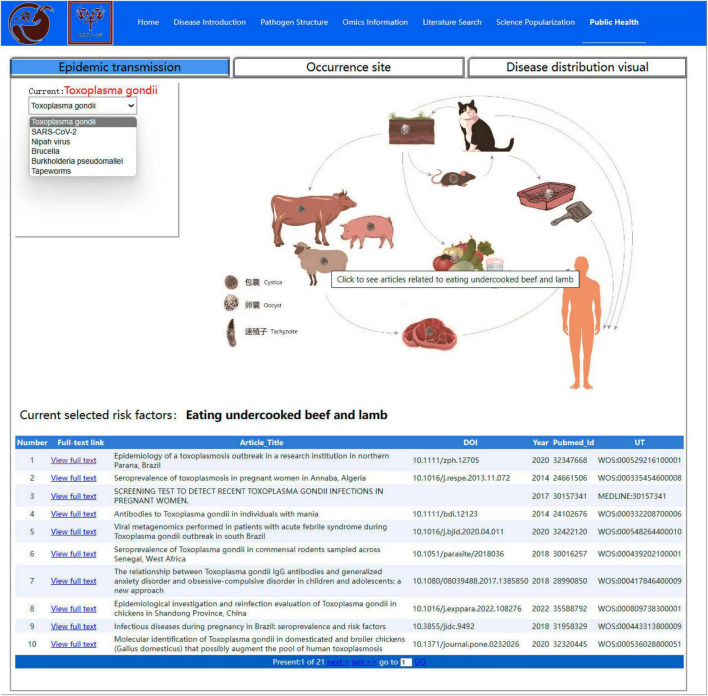
Risk factor interface in the public health module.

Presently, the public health module contains three sub-modules: risk factors, disease distribution, and prevention and control. The Risk Factor submodule summarizes the risk factors of various zoonoses and visualizes them using elements such as pathogens, intermediate hosts, and terminal hosts, thereby visually showing the transmission route of infectious diseases. There are various possible transmission routes for *Toxoplasma gondii*, including accidental ingestion of infected eggs from contaminated soil, water, raw vegetables, and fruits (Figure 7). The Risk Factor submodule also classifies the references containing each risk element, and the literature source can be traced by clicking on that element. When users click on animal elements (such as cattle and sheep) the page displays literature reports on the transmission of *Toxoplasma gondii* through cattle and sheep.

**FIGURE 8:**
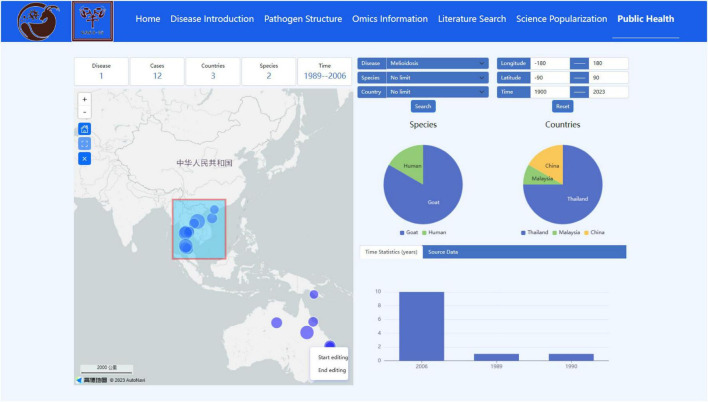
Disease distribution interface in the public health module.

The Disease Distribution visual interface provides a view of the latest reports of global infectious disease outbreaks and geolocation on Amap, and provides multidimensional search fields. The gadgets on the map can be zoomed in and out of the window to select the outbreak area. For example, the system calculates the countries, species, number of disease outbreaks, time span and other information involved in the *Burkholderia pseudomallei* outbreak in a selected region through background analysis of the website, and visualizes the statistical information in various forms such as pie charts, bar charts, and tables (Figure 8). In addition, the raw data for analysis can be downloaded for users to consult and analyze their own related research.

### 3.3 Other functions

The SGPD also provides easy access to other databases. Users can access nucleic acid databases, literature retrieval databases, and other scientific research tools to expand the visibility and accessibility of research findings. In addition, the platform provides data upload and export ports that can realize data updates and resource sharing.

## 4 Discussion

An increasing number of sheep and goat pathogens were identified and isolated over the past few decades. Unfortunately, a lack of awareness of these pathogens, epidemics, and inadequate protective measures have led to their further spread. Comprehensive pathogen data play an important role in clinical diagnosis, public health investigations, and outbreak surveillance (Gambarini et al., 2022). However, data resources suffer from redundancy, complexity, fragmentation, and lack of specialization (Wu et al., 2013; Zeng et al., 2022). Moreover, there is a lack of interdisciplinary analysts specializing in microbiology, bioinformatics, and public health in most laboratories. This limits the application of pathogens in epidemic prevention and control. Therefore, the SGPD was developed as a sheep and goat pathogen information retrieval system to enable the integration and sharing of sheep and goat pathogen data. This is the first database consisting of a comprehensive platform integrating multiple functions to systematically present the pathogenesis, histopathology, epidemiology, genomics, transcriptomics, and public health of sheep and goat infectious diseases that offers important research and preventive and control measures. The SGPD integrates data from large-scale authoritative websites and experimental studies, and its hunger for data has shined published data again. This is also the first step in the systematic collection and study of sheep and goat pathogens.

The most prominent way to obtain information on sheep and goat pathogens during the data collection of the SGPD was to search the scientific literature, with the exception of several specialized data platforms and monographs. Publishing literature is an important means for researchers worldwide to share their results. We extracted the valuable and neglected information from scientific literature. Finally, this database can be accessed by textual queries and visualizing text. This comprehensive literature search takes full account of the scientific research and practical applications. For example, pathogen identification often required an electron microscopy diagram of the pathogen in the past as a reference for morphology. Ultimately, the strain subspecies are determined after calculating biochemical indices, amplifying PCR, and sequencing 16S rRNA (Cao et al., 2018b; Suárez-Esquivel et al., 2020). Therefore, publicly available electron micrographs and tissue sections of sheep and goat pathogens in the scientific literature were collected using the Pathogen Structure module. This is intended to serve as a quick reference tool for pathogen identification.

The occurrence and prevalence of sheep and goat epidemics have seriously constrained the development of the sheep and goat breeding industry within the intensive mode of breeding, while zoonotic epidemics have seriously jeopardized public safety (Barbour et al., 1997; Judson and Rabinowitz, 2021). One global strategy (“One Health”) proposed that critical public health issues (Jiang and Jiang, 2018; Di Lorenzo et al., 2023) are closely linked between human, animal, and environmental health. The Public Health module of this database was designed to utilize multilatitude data to provide a more intuitive picture of the complex and unified relationship between people, animals, and the environment based on the “One Health” concept. For example, the Risk Factor submodule shows (Figure 7) that *Toxoplasma gondii* transmission through cat infection is a major mode of transmission (Dubey et al., 2020), whereas illnesses in cattle and sheep and goat fed contaminated grasses may be overlooked, which serves as a very important warning to users. People should be cautious about eating raw beef and lamb to prevent accidental ingestion of infectious eggs of *Toxoplasma gondii*. In addition, the Disease Distribution submodule enabled a bidirectional channel for data retrieval and visual analysis, thus maximizing the distribution of data availability. It is well known that melioidosis is a zoonotic epidemic prevalent in the tropics, with pathogenic bacteria (*Burkholderia pseudomallei*) present in the soil and water (Chantratita et al., 2023). *Burkholderia pseudomallei* was mainly distributed in Southeast Asia, Oceania, South Africa, and other regions within the 33 °S and 47 °N latitudes according to the Disease Distribution submodule (Figure 8). This range has a humid climate with moist soil that creates favorable conditions for the incidence and spread of *Burkholderia pseudomallei*. In China, there has been a particular concentration of human cases of melioidosis in the tropical province of Hainan, and a strain of *Burkholderia pseudomallei* of sheep and goat origin was isolated by our laboratory team (Cao et al., 2018a). Therefore, surveillance can focus on specific risk areas based on a good understanding of the risk factors and the regional distribution of epidemics. For example, it is necessary to conduct detailed outbreak investigations of human outbreaks of melioidosis, strengthen surveillance of outbreaks among livestock (especially flocks), and monitor the environmental sanitation of soil and water in Hainan Island, China.

The SGPD is a comprehensive database containing text descriptions, images, videos, schematics, and documents. A variety of data were adopted, and the presentation of data was increased in the process of creating the SGPD in an attempt to satisfy the majority of sheep and goat pathway-related research and applications owing to the dispersed sheep and goat pathogen data, the relative difficulty of obtaining information, and the different needs of researchers. Subsequent versions of the SGPD will provide more predefined workflows for pathogen identification and comprehensive analyses, including guidelines for sampling and sequencing, raw read preprocessing, and database annotation. Users are encouraged to upload the original experimental data to the database by using the “Data Upload” function of the database to share the research results with more researchers.

## 5 Conclusion

The SGPD is a comprehensive information retrieval and data analysis platform for sheep and goat pathogens. The main purpose of constructing the SGPD is to promote the effective integration and management of datasets. Currently, the SGPD provides six functional modules: disease introduction, pathogen structure, omics information, literature search, scientific popularization, and public health. The SGPD can be upgraded into a global data retrieval and analysis platform with the generation and integration of more comprehensive sheep and goat pathogen data.

## Data availability statement

The original contributions presented in the study are included in the article/Supplementary material, further inquiries can be directed to the corresponding authors.

## Author contributions

HP: Writing – original draft, Data curation, Investigation, Methodology, Software. ZJ: Writing – review and editing, Data curation. HL: Data curation, Writing – review and editing. SuL: Data curation, Writing – review and editing. LX: Data curation, Writing – review and editing. ShL: Data curation, Writing – review and editing. YM: Data curation, Writing – review and editing. YF: Data curation, Writing – review and editing. TC: Data curation, Writing – review and editing. QC: Funding acquisition, Project administration, Supervision, Writing – review and editing. SC: Funding acquisition, Project administration, Supervision, Writing – review and editing. LD: Funding acquisition, Project administration, Supervision, Writing – review and editing. CM: Funding acquisition, Project administration, Supervision, Writing – review and editing. FW: Funding acquisition, Project administration, Supervision, Writing – review and editing. HG: Funding acquisition, Project administration, Supervision, Writing – review and editing.

## References

[B1] Barbour E. K. Nabbut N. H. Hamadeh S. K. Al-Nakhli H. M. (1997). Bacterial identity and characteristics in healthy and unhealthy respiratory tracts of sheep and calves. *Vet. Res. Commun.* 21 421–430. 9266661 10.1023/a:1005855318665

[B2] Cao R. Y. Zhang Z. X. Nie X. Li B. B. Huang H. F. Yang X. J. (2018a). Isolation and characterization of Burkholderia cepacia of sheep origin. *J. Inner Mongolia Agric. Univ*. 39, 1–6 (In Chinese).

[B3] Cao R. Y. Zhang Z. X. Nie X. Li B. B. Huang H. F. Yang X. J. (2018b). Isolation and identification of Pasteurella multocida and its phylogenetic analysis. *Chinese J. Vet. Med*. 54, 55—58 (In Chinese).

[B4] Cao X. Li Z. Liu Z. Fu B. Liu Y. Shang Y. (2018). Molecular epidemiological characterization of Brucella isolates from sheep and yaks in northwest China. *Transbound. Emerg. Dis.* 65 e425–e433 (In Chinese). 29193808 10.1111/tbed.12777

[B5] Chantratita N. Phunpang R. Yarasai A. Dulsuk A. Yimthin T. Onofrey L. A. (2023). Characteristics and one year outcomes of melioidosis patients in Northeastern Thailand: a prospective, multicenter cohort study. *Lancet Reg. Health Southeast Asia* 9:100118. 10.1016/j.lansea.2022.100118 PMC9788505 36570973

[B6] Di Lorenzo A. Mangone I. Colangeli P. Cioci D. Curini V. Vincifori G. (2023). One health system supporting surveillance during COVID-19 epidemic in Abruzzo region, southern Italy. *One Health* 16:100471 (In Chinese). 10.1016/j.onehlt.2022.100471 PMC9726647 36507072

[B7] Dubey J. P. Cerqueira-Cézar C. K. Murata F. H. A. Kwok O. C. H. Yang Y. R. Su C. (2020). All about toxoplasmosis in cats: the last decade. *Vet. Parasitol.* 283:109145. 10.1016/j.vetpar.2020.109145 32645556

[B8] Galante D. Cafiero M. A. Raele D. A. Pugliese N. Padalino I. Cavaliere N. (2019). Identification and characterization of Orf viruses isolated from sheep and goats in Southern Italy. *Vet. Ital.* 55 347–353. 31955557 10.12834/VetIt.1025.5477.2

[B9] Gambarini V. Pantos O. Kingsbury J. M. Weaver L. Handley K. M. Lear G. (2022). PlasticDB: a database of microorganisms and proteins linked to plastic biodegradation. *Database* 2022:baac008. 10.1093/database/baac008 PMC9216477 35266524

[B10] Gao S. B. Wei X. J. Liu A. L. Sun L. K. Wang Y. M. (2022). Construction of a method for analyzing direct economic losses and cost-effectiveness of prevention and control of brucellosis in sheep and empirical research. *China Anim. Health Inspection* 39, 1–6 (In Chinese).

[B11] Han B. C. Wei X. N. Zhang J. X. Truong N. Q. T. Westgate C. L. Zhao R. Y. (2013). MicrobPad MD: Microbial pathogen diagnostic methods database. *Infect. Genet. Evol.* 13 261–266. 23178820 10.1016/j.meegid.2012.10.017

[B12] Hawwas H. A. E. Aboueisha A. M. Fadel H. M. El-Mahallawy H. S. (2022). *Salmonella* serovars in sheep and goats and their probable zoonotic potential to humans in Suez Canal Area. *Egypt. Acta Vet. Scand.* 64:17. 10.1186/s13028-022-00637-y PMC9336019 35906669

[B13] Jiang P. Jiang Q. Y. (2018). The development of the concept of “One Health” and its contemporary value. *J. Dialectics Nat*. 40, 17–22 (In Chinese).

[B14] Judson S. D. Rabinowitz P. M. (2021). Zoonoses and global epidemics. *Curr. Opin. Infect. Dis.* 34 385–392. 34310453 10.1097/QCO.0000000000000749

[B15] Lange R. E. Dupuis, Ii A. P. Prusinski M. A. Maffei J. G. Koetzner C. A. (2023). Identification and characterization of novel lineage 1 Powassan virus strains in New York State. *Emerg. Microbes Infect.* 12:2155585. 10.1080/22221751.2022.2155585 PMC9788702 36503411

[B16] Leandro A. S. de Castro W. A. C. Lopes R. D. Delai R. M. Villela D. A. M. de-Freitas R. M. (2022). Citywide integrated *Aedes aegypti* mosquito surveillance as early warning system for Arbovirus transmission, Brazil. *Emerg. Infect. Dis.* 28 701–706. 10.3201/eid2804.211547 PMC8962889 35318912

[B17] Mugezi I. Kimaanga M. Namwabira A. Chevanne E. Nekouei O. McLaws M. (2020). Risk of foot and mouth disease spread through cattle movements in Uganda. *Rev. Sci. Tech.* 39 847–861. 35275131 10.20506/rst.39.3.3182

[B18] Omodo M. Gardela J. Namatovu A. Okurut R. A. Esau M. Acham M. (2023). Anthrax bio-surveillance of livestock in Arua district. Uganda, 2017-2018. *Acta Trop.* 240:106841. 10.1016/j.actatropica.2023.106841 36693517

[B19] Ouchene N. Hamidović A. Khelifi Touhami N. A. Aroussi A. Ouchetati I. Khelef D. (2023). Seroprevalence and risk factors of Toxoplasma gondii infection in sheep in Algeria. *Comp. Immunol. Microbiol. Infect. Dis.* 95:101960. 10.1016/j.cimid.2023.101960 36963357

[B20] Simões J. Abecia J. A. Cannas A. Delgadillo J. A. Lacasta D. Voigt K. (2021). Review: Managing sheep and goats for sustainable high yield production. *Animal* 15 (Suppl. 1):100293. 10.1016/j.animal.2021.100293 34294548

[B21] Stella E. Mari L. Gabrieli J. Barbante C. Bertuzzo E. (2020). Permafrost dynamics and the risk of anthrax transmission: a modelling study. *Sci. Rep.* 10:16460. 10.1038/s41598-020-72440-6 PMC7541526 33028874

[B22] Stelzer S. Basso W. Benavides Silván J. Ortega-Mora L. M. Maksimov P. Gethmann J. (2019). Toxoplasma gondii infection and toxoplasmosis in farm animals: risk factors and economic impact. *Food Waterborne Parasitol.* 15:e00037. 10.1016/j.fawpar.2019.e00037 PMC7033994 32095611

[B23] Suárez-Esquivel M. Hernández-Mora G. Ruiz-Villalobos N. Barquero-Calvo E. Chacón-Díaz C. Ladner J. T. (2020). Persistence of Brucella abortus lineages revealed by genomic characterization and phylodynamic analysis. *PLoS Negl. Trop. Dis.* 14:e0008235. 10.1371/journal.pntd.0008235 32287327 PMC7182279

[B24] Wang X. Liu Z. Li X. Li D. Cai J. Yan H. (2020). SPDB: a specialized database and web-based analysis platform for swine pathogens. *Database* 2020:baaa063. 10.1093/database/baaa063 32761141 PMC7409514

[B25] Wu L. Sun Q. Sugawara H. Yang S. Zhou Y. McCluskey K. (2013). Global catalogue of microorganisms (gcm): a comprehensive database and information retrieval, analysis, and visualization system for microbial resources. *BMC Genom.* 14:933. 10.1186/1471-2164-14-933 24377417 PMC3890509

[B26] Zeng J. Tu Q. Yu X. Qian L. Wang C. Shu L. (2022). PCycDB: a comprehensive and accurate database for fast analysis of phosphorus cycling genes. *Microbiome* 10:101. 10.1186/s40168-022-01292-1 35787295 PMC9252087

[B27] Zhang Y. Mi R. Xie J. Jia H. Ling H. Zhang X. (2022). Seroprevalence and the risk factor of Toxoplasma gondii Infection to Slaughter Pigs in Chongqing, China. *Vector Borne Zoonotic Dis.* 22 238–243. 35404131 10.1089/vbz.2021.0101

[B28] Zhou S. Y. Liu B. Han Y. L. Wang Y. Y. Chen L. H. Wu Z. Q. (2022). ZOVER: the database of zoonotic and vector-borne viruses. *Nucleic Acids Res.* 50 D943–D949. 34634795 10.1093/nar/gkab862 PMC8728136

