# Supplementary material for: Sheep and goat pathogen database: a pathogen data integration and analysis database of sheep and goat infectious diseases
Source: Front Microbiol. 2024 Jan 12;14:1299303. doi: 10.3389/fmicb.2023.1299303 (PMC10811017; doi:10.3389/fmicb.2023.1299303)
Supplement: Supplementary file 1 [file Table_1.DOCX]

Supplementary Material

Sheep and Goat Pathogen Database: a pathogen data integration and analysis database of sheep and goat infectious diseases

Haoju Pan, Zizhuo Jiao, Hong Li, Suya Li, Le Xu, Shiyuan Li, Yong Meng, Yujing Fu, Taoyu Chen, Qiaoling Chen, Si Chen, Li Du, Churiga Man, Fengyang Wang*, Hongyan Gao*

Hainan Key Laboratory of Tropical Animal Reproduction & Breeding and Epidemic Disease Research, Animal Genetic Engineering Key Laboratory of Haikou, School of Tropical Agriculture and Forestry, Hainan University, Haikou 570228, People's Republic of China.

*** Correspondence:**Fengyang Wang

E-mail: fywang68@163.com

Hongyan Gao

E-mail: gaohongyan@hainanu.edu.cn

Table S1 Descriptions of 44 sheep and goat pathogens in the database

| **No.** | **Classification** | **Pathogen** | **Infectious Disease** | **Whether Zoonosis** |
| --- | --- | --- | --- | --- |
| 1 | Bacteria | *Brucella* | Brucellosis | Yes |
| 2 | Bacteria | *Mycobacterium tuberculosis* | Tuberculosis | Yes |
| 3 | Bacteria | *Bacillus anthracis* | Anthrax | Yes |
| 4 | Bacteria | *Clostridium perfringens* | Clostridium perfringens infection | No |
| 5 | Bacteria | *Escherichia coli* | Colibacillosis | No |
| 6 | Bacteria | *Pasteurella multocida* | Pasteurellosis | No |
| 7 | Bacteria | *Streptococcus* | Streptococcus infection | No |
| 8 | Bacteria | *Mannheimia haemolytica* | Mannheimia haemolytica infection | No |
| 9 | Bacteria | *Mycobacterium avium ssp paratuberculosis* | Paratuberculosis | No |
| 10 | Bacteria | *Eperythrozoon* | Eperythrozoonosis | No |
| 11 | Bacteria | *Salmonella* | Salmonellosis | Yes |
| 12 | Bacteria | *Listeria* | Listeriosis | Yes |
| 13 | Bacteria | *Burkholderia pseudomallei* | Melioidosis | Yes |
| 14 | Bacteria | *Coxiella burnetii* | Q fever | Yes |
| 15 | Bacteria | *Leptospira* | Leptospirosis | Yes |
| 16 | Bacteria | *Corynebacterium pseudotuberculosis* | Caseous lymphadenitis | No |
| 17 | Bacteria | *Anaplasma ovis* | Anaplasmosis | No |
| 18 | Virus | Foot-and-mouth disease virus | Foot-and-mouth disease | No |
| 19 | Virus | Nipah virus | Nipah virus infection | Yes |
| 20 | Virus | Prion | Scrapie | No |
| 21 | Virus | Peste des petits ruminants virus | Peste des petits ruminants | No |
| 22 | Virus | Bluetongue virus | Bluetongue | No |
| 23 | Virus | Pseudorabies virus | Pseudorabies | No |
| 24 | Virus | Rabies virus | Rabies | Yes |
| 25 | Virus | Japanese encephalitis virus | Japanese encephalitis | Yes |
| 26 | Virus | Sheep poxvirus/Goat poxvirus | Sheep and goat pox | No |
| 27 | Virus | Rotavirus | Rotavirus infection | No |
| 28 | Virus | Caprine arthritis encephalitis virus | Caprine arthritis encephalitis | No |
| 29 | Virus | Visna/Maedi virus | Maedi-Visna disease | No |
| 30 | Virus | Jaagsiekte sheep retrovirus | Sheep pulmonary adenomatosis | No |
| 31 | Virus | Orf virus | Contagious ecthyma | No |
| 32 | Virus | SARS-CoV-2 | COVID-19 | Yes |
| 33 | Parasite | *Schistosoma japonicum* | Schistosomiasis japonica infection | No |
| 34 | Parasite | Tapeworms | Echinococcosis | Yes |
| 35 | Parasite | *Toxoplasma gondii* | Toxoplasmosis | Yes |
| 36 | Parasite | *Orientobilharzia* | Orientobilharziasis | No |
| 37 | Parasite | *Haemonchus contortus* | Haemonchus contortus infection | No |
| 38 | Parasite | *Trypanosoma evansi* | Trypanosoma evansi infection | No |
| 39 | Parasite | *Cryptosporidium* | Cryptosporidiosis | No |
| 40 | Parasite | *Fasciola hepatica* | Fascioliasis | Yes |
| 41 | Parasite | *Babesia motasi* | Piroplasmosis | No |
| 42 | Parasite | *Leishmania* | Leishmaniasis | Yes |
| 43 | *Chlamydia* | *Chlamydia trachomatis* | Chlamydia disease | No |
| 44 | *Mycoplasma* | *Mycoplasma capricolum* subsp. *capripneumoniae* | Contagious caprine pleuropneumonia | No |
